# Effects of a Glycosylated Form of Active Vitamin D Combined with Natural Triterpenes on Sow Productive Performance, Mineral Homeostasis, Immune Biomarkers and Serum Proteome

**DOI:** 10.3390/vetsci13030246

**Published:** 2026-03-05

**Authors:** Luca Marchetti, Raffaella Rebucci, Carlotta Giromini, Elisa Margherita Maffioli, Gabriella Tedeschi, Valentino Bontempo

**Affiliations:** Department of Veterinary Medicine and Animal Sciences (DIVAS), University of Milan, Via dell’ Università 6, 26900 Lodi, Italyelisa.maffioli@unimi.it (E.M.M.); gabriella.tedeschi@unimi.it (G.T.); valentino.bontempo@unimi.it (V.B.)

**Keywords:** vitamins, vitamin D, gestation, lactation, pigs, nutrition, feed, calcium, minerals, proteome

## Abstract

This study evaluated the effects of the dietary administration of a glycosylated form of active vitamin D (calcitriol, 1,25(OH)_2_D_3_) combined with ursolic acid (UA) and oleanolic acid (OA) triterpenes on sow health and productivity. Twenty-four third-parity Landrace × Large White sows were allocated at day 108 of gestation into three groups: a control group receiving 1800 IU/kg of vitamin D_3_, and two treatment groups receiving the control diet supplemented with either 0.64 µg/kg (ACTD1) or 0.96 µg/kg (ACTD2) of glycosylated 1,25(OH)_2_D_3_ plus 140 or 210 µg/kg of UA + OA (4:1 ratio), respectively. Diets were administered from late gestation through the end of lactation. Farrowing duration, sow body weight, backfat thickness, and litter growth were recorded. Blood samples collected at key physiological stages were analyzed for pro-inflammatory cytokines, mineral homeostasis, endocrine markers, and serum proteome. Farrowing time was reduced in both treatment groups compared with the control (*p* < 0.05). Treated sows exhibited lower backfat thickness at the end of lactation and improved litter weights at farrowing, after cross-fostering, and at weaning (*p* < 0.05). Plasma pro-inflammatory cytokines (TNF-α, IL-1α, and IL-1β) were reduced at the end of lactation in ACTD1 and ACTD2 sows, with TNF-α and IL-1β already decreased after farrowing (*p* < 0.05). Treated sows also displayed decreased plasma parathormone concentrations at the end of lactation, along with increased circulating 1,25(OH)_2_D_3_ and calcium concentrations after farrowing and at lactation end (*p* < 0.05), while plasma phosphate levels remained unchanged. Proteomic analysis supported the systemic availability of the supplemented compounds and their involvement in metabolic and inflammatory pathways rather than calcium transport or vitamin D binding mechanisms. Overall, this nutritional strategy influenced the immune modulation while maintaining mineral homeostasis via modest endocrine adaptations. Larger-scale trials are warranted to confirm these results and to evaluate their practical applicability under commercial production conditions.

## Simple Summary

This study evaluated whether adding a more bioavailable form of vitamin D, together with two natural plant-derived compounds (ursolic acid and oleanolic acid), to sow diets could improve their health and performance. Twenty-four sows in late pregnancy were divided into three groups. One group received a normal diet, while the other two groups received the same diet plus different amounts of the vitamin D and plant compound mixture. The results showed clear benefits for the supplemented sows. Both treated groups had a shorter and easier farrowing process compared with the control group. Piglets born from treated sows were heavier at birth and at weaning, showing better growth. Analyses showed that treated sows had lower levels of substances linked to inflammation. The supplements also improved calcium balance. Additional analyses confirmed that the supplemented compounds were absorbed and active in the body. Overall, adding this vitamin D and plant-based supplement to sow diets during late pregnancy and lactation helped improve sow health and supported better growth of piglets.

## 1. Introduction

Vitamin D is commonly present in livestock feed as cholecalciferol (vitamin D_3_) [1]. Cholecalciferol can be self-synthesized by UVB exposure from 7-dehydrocholesterol, which represents an intermediate of cholesterol synthesis [2]. However, modern farming practices limit the exposure to natural sunlight, which decreases the availability of self-synthesized vitamin D [3].

Once ingested, dietary cholecalciferol requires two subsequent hydroxylation processes in the liver and kidneys to become biologically active. In particular, after twenty-five-and-one-position hydroxylation in the liver and kidneys, calcitriol (1,25(OH)_2_D_3_) can interact with vitamin D receptors (VDRs), which are present both in digestive and reproductive organs [4]. Hence, one of the main biological functions of calcitriol is to maintain calcium (Ca) homeostasis and its absorption and reabsorption in the intestine and kidneys [5].

On the other hand, 1,25(OH)_2_D_3_ has a strong influence on innate and adaptive immunity pathways thanks to its interactions with VDRs. More specifically, in different animal models, 1,25(OH)_2_D_3_ has been reported to ameliorate the pro-inflammatory response, favoring the inhibition of TNF-α serum levels [6].

Nonetheless, hyperprolific sows are susceptible to vitamin D and Ca deficiency during the last period of gestation and throughout lactation [7,8]. These phases are characterized by increasing Ca requirements to sustain both colostrum and milk production and offspring skeletal muscle development as well [9]. Vitamin D and calcium deficiencies in hyperprolific sows can adversely affect parturition, piglet survival, and maternal health [10]. Ca is critical for effective myometrial contractions, and hypocalcemia around farrowing is associated with prolonged parturition, increased stillbirths, and asphyxia in piglets due to weak uterine contractions and delayed delivery intervals. Hence, it is clear that nutritional strategies aiming to enhance calcium availability can reduce stillborn piglets and improve piglet viability at birth. Indeed, adequate vitamin D status enhances calcium absorption and skeletal development in piglets and supports maternal bone health and metabolic function [11,12,13]. Thus, maintaining sufficient vitamin D and calcium is essential to optimize farrowing efficiency, reduce perinatal losses, and support maternal recovery.

However, when considering modern hyperprolific sows, large litters can be negatively affected by limited colostrum intake, resulting in poor growth performance and penalized active and passive immune functions [14]. Indeed, it was previously reported that reducing maternal pro-inflammatory cytokines could be associated with higher live-born weights, which comprehensively affects future offspring growth [15].

Guaranteeing ideal vitamin D intake and the bioavailability of 1,25(OH)_2_D_3_ could favor the maintenance of electrolyte homeostasis, protecting sows from Ca imbalances, maintaining immune functions and reducing offspring mortality. For these reasons, in the past, dietary vitamin D_3_ requirements for sows were remodulated and increased from 200 to 800 international units per kg of complete feed [16,17]. However, subsequent evaluations indicated that a greater dose of vitamin D_3_ (1400 IU/kg of complete feed) was needed to sustain the reproductive performance of sows [18]. Nowadays, in Europe, vitamin D_3_ can be supplemented in pig feed by up to 2000 international units per kg of complete feed [19]. Nonetheless, the possibility of optimizing vitamin D utilization through the dietary administration of vitamin D_3_, along with active vitamin D forms, in pig diets is a topic of great interest [20]. Indeed, unlike cholecalciferol, 1,25(OH)_2_D_3_ bypasses liver and kidney hydroxylation steps, ensuring higher functional efficiency per unit administered [20,21]. Hence, it directly stimulates the VDR-mediated expression of intestinal calcium transport proteins, thereby enhancing calcium absorption during periods of high demand [21,22]. Reasonably, this direct hormonal action can contribute to the greater metabolic stability of calcium and phosphate homeostasis when endogenous conversion capacity may be limited. Thus, the described nutritional strategy may enhance the bioavailability and biological activity of circulating 1,25(OH)_2_D_3_, thereby improving calcium homeostasis and sow immune function, with potential carry-over benefits on offspring growth.

Glycosylated 1,25(OH)_2_D_3_ represents a more bioavailable and soluble form of the self-synthesized molecule. 1,25(OH)_2_D_3_ glycosides are commonly present in different plants, such as *Solanum glaucophyllum*, *Trisetum flavescens*, *Cestrum diurnum* and *Nicotiana glauca* [23]. To become biologically active and stimulate Ca metabolism, glycosylated 1,25(OH)_2_D_3_ requires cleavage by bacteria with β-glycosidase activity, which normally occurs in the large intestine of monogastric animals [24]. On the other hand, the combination of glycosylated 1,25(OH)_2_D_3_ with phytocompounds has been evaluated in recent studies. These solutions are known for their capacity to improve the reproductive performance of sows [25].

Triterpenes are an extended group of plant metabolites whose primary role is to protect the host from pathogens and parasites [26]. In particular, ursolic acid (UA) and oleanolic acid (OA) are triterpenic acids characterized by diversified antibacterial and antiparasitic properties [27]. UA is a ursane-type pentacyclic triterpenoid isolated from a wide variety of plants, such as *Rosmarinus officinalis* L., *Origanum majorana* L., *Thymus vulgaris* L., *Malus domestica* Borkh., *Lavandula angustifolia* Mill., and *Sambucus nigra* L. [28]. OA is a pentacyclic triterpenoid found in the *Oleaceae*, *Rosaceae*, *Araliaceae*, and *Caricaceae* plant families [29]. Both UA and OA have been indicated as effective immunomodulators in previous in vivo and in vitro studies.

The theoretical synergy between ursolic acid (UA) and oleanolic acid (OA) with glycosylated 1,25(OH)_2_D_3_ derives from complementary biological actions. UA and OA exert anti-inflammatory and immunomodulatory effects mainly through inhibition of NF-kB signaling, pathways that intersect with VDRs-mediated immune regulation [21,30,31,32]. In addition, OA has been shown to improve intestinal barrier integrity and may facilitate the absorption and stability of lipophilic compounds, including vitamin D metabolites [32]. Glycosylation of 1,25(OH)_2_D_3_ may further enhance molecular stability and prolong VDR activation. Collectively, these mechanisms provide a plausible basis for synergistic effects on immune function and calcium metabolism.

Hence, the combined action of plant-derived triterpenes and glycosylated 1,25(OH)_2_D_3_ may be particularly relevant during the late-gestation-to-lactation transition in hyperprolific sows; however, studies specifically addressing this critical window are lacking. In addition, the mechanistic basis of their potential synergistic effects has not been elucidated, and dose–response data for their combined dietary inclusion remain unavailable.

Therefore, the present study aimed to evaluate the effects of dietary administration of glycosylated calcitriol combined with the triterpenes ursolic acid and oleanolic acid in third-parity sows. Specifically, the study investigated the impact of this nutritional strategy on productive performance, systemic immune status, and metabolic responses associated with the 1,25(OH)_2_D_3_–parathyroid hormone axis, with particular emphasis on calcium and inorganic phosphate homeostasis.

## 2. Materials and Methods

### 2.1. Ethical Statement

The protocol of the present study was evaluated and approved by the Animal Welfare Committee of the University of Milan (approval code OPBA_55_2023, 16 June 2023).

### 2.2. Experimental Design and Animals Housing

The present study was performed on a commercial farm located in northern Italy (Azienda Agricola Arioli-Sangalli, Genzone, Italy).

The sample size for the present trial was determined a priori based on the primary metabolic variables of interest (vitamin D metabolites). Sample size was calculated a priori, considering a one-way ANOVA with three treatments using a noncentral F distribution. Assuming a significance level of α = 0.05, a statistical power of 0.80, and a large effect size (Cohen’s f = 0.40), the noncentrality parameter was defined as λ = f^2^ × *n*. Numerical solution of the power equation indicated that 8 sows per treatment group (total n = 24) were required. Calculations were confirmed using G*Power version 3.1.9.7 (Heinrich-Heine-University Düsseldorf, Düsseldorf, Germany).

Hence, twenty-four third-parity Topigs Norsvin TN70 Landrace x Large White sows (320.09 ± 26.22 kg) were selected seven days before the predicted farrowing date (day 108 of gestation) and allocated into individual farrowing crates (2.20 × 1.80 m) equipped with single feeders until the end of lactation. Gestating sows were divided into 3 experimental groups, with 8 animals per treatment, and distributed between two farrowing rooms, each with a maximum capacity of 12 farrowing crates (two rows of 6 crates for each room). A randomized block design based on body weight was considered for sow distribution. The control group (CTR) was fed a standard basal diet formulated to meet the nutrient requirements of gestating and lactating sows [17] (Table 1), supplemented with a vitamin and mineral premix providing 1800 IU of cholecalciferol per kg of diet (feed-grade, ≥98% purity, and in accordance with European Regulation 1831/2003).

The treatment 1 group (ACTD1) received the CTR diet supplemented with 400 mg/kg of a premix composed of golden oat grass meal, providing 0.64 µg/kg of glycosylated 1,25(OH)_2_D_3_ and 140 µg/kg of a combination of UA and OA triterpenes. The treatment 2 group (ACTD2) received the CTR diet supplemented with 600 mg/kg of the same premix, providing 0.96 µg/kg of glycosylated 1,25(OH)_2_D_3_ and 210 µg/kg of UA + OA triterpenes. The treatment product (Active D) was provided by Phytobiotics Futterzusatzstoffe GmbH (Wallufer Str.10a, 65343 Eltville, Germany) and consisted of a premix composed of golden oat grass meal, characterized by a content of 1.60 mg/kg of glycosylated calcitriol and 350 mg/kg of UA + OA (4:1 ratio). From the entrance into individual crates until parturition, sows were fed 2.8 kg of feed per day. Immediately after farrowing, the amount of feed gradually increased from 2.9 kg to 8 kg/d (day 16 after farrowing), which was maintained until the end of lactation. Feed was diluted in water at a 1:2.8 ratio before automatic distribution at 10 AM and 2 PM. The treatment compound was administered on top of the feed immediately after distribution. Sows had free access to water.

The average temperature of the farrowing room was 25 °C; nest areas for piglets with heat lamps (250 W) and paper shavings were prepared in each farrowing crate before trial start. Litters had creep-feed and water at disposal starting from the first day of life. Farm personnel routinely checked the health of sows and piglets.

Farrowing was not synchronized. Cross-fostering was performed exclusively among sows within the same experimental group (CTR, ACTD1, or ACTD2) and was allowed up to 7 days post-partum, following standardized farm management practices. Piglets were selected for cross-fostering primarily to equalize litter size. Rescue decks were also used according to routine procedures. The combined use of cross-fostering and rescue decks within each experimental group ensured that only piglets from enrolled litters were included, allowed for the standardization of litter size, and minimized potential effects of external piglets on within-litter body weight development. Hence, the inclusion of piglets deriving from litters of sows not involved in the study was avoided, resulting in an average number of 10.71 piglets per sow after the cross-fostering period. Assistance was provided to sows during farrowing through trained farm personnel.

### 2.3. Feed Analyses

Feed samples were collected before trial start to determine primary nutritional components (dry matter, ash, crude fiber, crude protein, ether extract) according to AOAC methods [33]. Dry matter (DM) was determined by drying the samples in a forced-air oven at 65 °C for 24 h (AOAC, 930.15). Ash content was measured following a 3 h incineration in a muffle furnace at 550 °C (AOAC, 942.05). Crude fiber (CF) was analyzed using the filtering bag method (AOCS, Ba 6a-05) [34]. Crude protein (CP) was determined by the Kjeldahl method, considering a factor of 6.25 for nitrogen-to-protein conversion (AOAC, 2001.11). Ether extract (EE) was measured by Soxtec extraction with petroleum ether (AOAC, 2003.05). Feed calcium (Ca) and phosphorus (P) levels were analyzed by using inductively coupled plasma-optical emission spectrometry (ICP-OES, Optima TM 8000, PerkinElmer, Shelton, WA, USA). Basal diet ingredients and composition are listed in Table 1.

### 2.4. Measurements and Samples Collection

The duration of farrowing (defined as the time from the expulsion of the first to the last piglet) was monitored using cameras (YI PRO 2K, 2304 × 1296P 30fps, YI Technology, Shanghai, China) installed at each corner of the farrowing rooms prior to the start of the experiment. Each camera was able to continuously and accurately monitor three sows. Body weights of sows were monitored by weighing the animals at the entrance in the farrowing room and at the end of lactation through a 250 × 70 cm electronic balance (CIMA control pig, CIMA, Correggio, Reggio Emilia, Italy). At trial start, within 12 h from parturition and at the end of lactation, sow backfat thickness was registered by a portable ultrasound echograph (Duo Scan:Go^TM^ Plus, IMV imaging, United Kingdom) at 65 mm on the right of the dorsal midline of the last rib. Immediately after farrowing, following the cross-fostering period (corresponding to 7 d of lactation) and at weaning, offspring were weighed using a 90 × 60 cm electronic balance (Soehnle Industrial Solutions GmbH, Backnang, Germany). At trial start, within 12 h from parturition and at the end of lactation, two blood samples were collected from the jugular vein of each sow by using 10 mL tubes armed with an 18 G needle (VACUETTE^®^, Merck KGaA, Darmstadt, Germany). In more detail, two different vacuum tubes were used for blood sampling. Specifically, red-top tubes without anticoagulants (Vacutainer Systems Europe, Meylan-Cedex, France) were used to collect blood aliquots for serum preparation, whereas green-top tubes containing sodium heparin (Vacutainer Systems Europe, Meylan-Cedex, France) were used to collect blood samples for plasma preparation. Blood samples were used to prepare plasma and serum following a centrifugation step [35,36]. Aliquots were stored at −20 °C until analysis.

### 2.5. Plasma Pro-Inflammatory Cytokines, Parathormone and Calcitriol Analyses

Plasma aliquots were used to determine pro-inflammatory cytokines. In particular, tumor necrosis factor-α (TNF-α), interleukin-1α (IL-1α), and interleukin-1β (IL-1β) were determined using commercial ELISA kit tests (Società Italiana Chimici s.r.l, Roma, Italy). Similarly, parathormone (PTH) and calcitriol (1,25(OH)_2_D_3_) plasma levels were evaluated through commercially available ELISA test kits (Società Italiana Chimici s.r.l, Roma, Italy) according to the manufacturer’s instructions [36,37]. For all the previous evaluations, 60 µL of plasma were extracted from each aliquot and diluted with a sample dilution buffer at a 1:2 ratio. After washing the plate 2 times, 50 µL of diluted sample was added to each well. Hence, 50 µL of Biotin-labeled Antibody working solution (Società Italiana Chimici s.r.l, Roma, Italy) was included, and the plate was incubated for 45 min at 37 °C. In total, 100 µL of HRP–Streptavidin conjugate (Società Italiana Chimici s.r.l, Roma, Italy) working solution was included. Following a second incubation period (30 min at 37 °C), the plate was washed 5 times. Then, 90 µL of TMB substrate solution (Società Italiana Chimici s.r.l, Roma, Italy) was added, followed by a static incubation for 15 min at 37 °C. Finally, 50 µL of stop solution was added to each well, and the plate was read at 450 nm using a microplate photometer (ScanReady P-800, Hangzhou LifeReal Biotechnology, Hangzhou, China). All the above parameters are expressed in pg/mL. Biological triplicates were considered for the analyses described.

### 2.6. Plasma Calcium and Phosphate Assessment

Plasma concentration of calcium (Ca) and phosphate (P) was assessed as described above, using inductively coupled plasma–optical emission spectrometry (ICP-OES, Optima TM 8000, PerkinElmer, Shelton, WA, USA) [38], following microwave digestion, as depicted by Chevallier et al. [39]. Ca and P plasma levels are expressed in mg/dL. Biological triplicates were considered for Ca and P evaluations.

### 2.7. Serum Proteomic Profile Evaluation

For mass spectrometry (MS) sample preparation, all serum samples were depleted for albumin using the Pierce Albumin Depletion Kit (Fisher Scientific GmbH, Schwerte, Germany), according to the manufacturer’s instructions. Protein concentration was determined by Bradford assay (Fisher Scientific GmbH, Schwerte, Germany) [40] before reduction, alkylation and in-solution tryptic digestion, as previously reported [41]. Peptides were desalted using Zip-Tip C18, as reported by Aletti et al. [42]. NanoHPLC coupled to MS/MS analysis was performed on a Dionex UltiMate 3000 directly connected to a Q-Exactive Plus Orbitrap mass spectrometer (Fisher Scientific GmbH, Schwerte, Germany) using a nanoelectrospray ion source. Peptide mixtures were enriched on a 75 μm ID × 150 mm EASY Spray PepMap RSLC C18 column (Fisher Scientific GmbH, Schwerte, Germany) and separated using the LC gradient: 1% acetonitrile (ACN) in 0.10% formic acid for 10 min, 1–5% ACN in 0.1% formic acid for 6 min, 5–38% ACN in 0.1% formic acid for 147 min, and 38–63% ACN in 0.10% formic for 3 min at a flow rate of 0.30 μL/min (Merck KGaA, Darmstadt, Germania). Flow-rate stability was ensured by the built-in pump control of the chromatographic system, which maintains a constant and reproducible flow throughout the analysis. During gradient elution, the column temperature was controlled and maintained at 35 °C [43]. Orbitrap MS spectra of eluting peptides were collected over an m/z range of 375–1500 at a resolution of 7000, operating in a data-dependent MS^2^ mode for the 10 most intense peaks from full scan using an isolation window of 3 m/z and a normalized collision energy of 35. HCD MS/MS spectra were acquired in Orbitrap at a resolution of 17,500. Dynamic exclusion was set to 30 s. Rejection of +1 and unassigned charge states was enabled.

The mass spectrometry proteomics data have been deposited in the ProteomeXchange Consortium via the PRIDE [44] partner repository (EMBL-EBI, Wellcome Genome Campus, Hinxton, Cambridgeshire, UK), with the data set identifier PXD070245.

**Figure 1:**
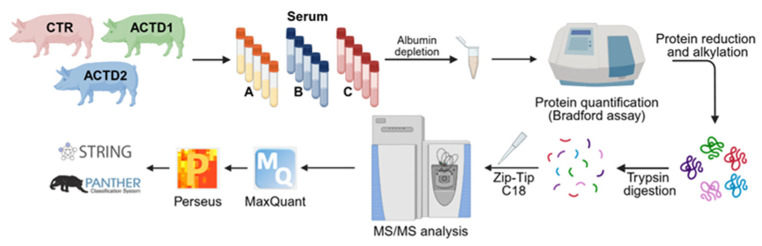
Shotgun label-free quantitative proteomic analysis. Overview of the workflow for the analysis of the effects of the described nutritional strategy on the serum proteome of sows at three depicted physiological stages: entry into the farrowing room, farrowing, and end of lactation.

A database search was conducted against the NBCI *Sus Scrofa* sequence database (released in 02/2025; 103,228 entries) with MaxQuant version 1.6.1.0 (Max-Planck-Institute of Biochemistry, Planegg, Germany). The initial maximum allowed mass deviation was set to 10 ppm for monoisotopic precursor ions and 0.5 Da for MS/MS peaks. Enzyme specificity was set to trypsin, defined as C-terminal to Arg and Lys, excluding Pro, and a maximum of two missed cleavages was allowed. Carbamidomethylcysteine was set as a fixed modification, while Met oxidation and Asn/Gln deamidation were set as variable modifications. Quantification in MaxQuant was performed using built-in label-free quantification (LFQ) algorithms based on the extracted ion intensity of precursor ions. The false discovery rate (1%) of the peptide-spectrum matches (PSMs) and protein identifications was estimated by searching MS/MS spectra against the corresponding reversed-sequence (decoy) database [45]. The analytical workflow is summarized in Figure 1.

### 2.8. Statistical and Bioinformatic Analyses

According to the sample size assessment reported above, experimental data concerning productive performance were analyzed through a one-way ANOVA using GraphPad Prism 9 9.3.1 (GraphPad Software Inc., SanDiego, CA, USA) to discriminate the differences among group means within each considered time point. Nonetheless, plasma parameters were analyzed through a two-way ANOVA to clearly describe the metabolic changes in the primary variables of interest over time. All the data collected were tested for normality through a Shapiro–Wilk test. In cases of non-normal distribution, a Kruskal–Wallis test was applied. Group comparisons were performed through a post hoc evaluation based on Tukey’s multiple comparisons test. The statistical model for blood parameters was the following:

*Y_ijkn_ = µ + Treatment_j_ + Time_k_ + (Treatment x Time)_jk_ + sow_n_ + e_ijkn_*

where *Yijkn* is the *Ith* recorded value for each considered parameter evaluated from sow *n* with *treatment j* (CTR: 0 mg/kg, ACTD1: 400 mg/kg, ACTD2: 600 mg/kg) at *time k* (entrance in the farrowing room, farrowing, end of lactation), while *eijkn* represents the residual error. µ is the intercept. Treatment and time were considered fixed effects, while the effect of the animal was accounted for as a random factor. Data collected at the first time point (entrance into the farrowing room) were used as covariates. The results are reported as mean ± standard error of the mean (SEM). Values are considered statistically significant for a confidence interval of 95% (*p* < 0.05) and highly significant for a confidence interval of 99% (*p* < 0.01).

The proteomic data analysis was performed using the Perseus software (version 1.5.5.3). Only the proteins present and quantified in at least 70% of the repeats were considered positively identified in a sample [46]. Focusing on specific comparisons (ACDT2 vs. ACDT1 and ACDT1 + ACDT2 vs. CTR), proteins were considered differentially expressed if they were present only in one condition or showed a significant t-test difference (Student’s test *p* < 0.05). One-way ANOVA followed by Bonferroni post hoc correction (*p* < 0.05) was carried out to identify proteins differentially expressed across all experimental groups.

Bioinformatic analyses were carried out using the Panther software, release 19.0 [47] (Health Sciences Library System, University of Pittsburgh, Pittsburgh, PA, USA) to classify the proteins of the various data sets and to cluster enriched annotation groups of biological processes (GOBPs), reactome pathways, and networks within the set of identified proteins. Functional grouping was based on Fisher’s exact test, *p* < 0.05. Interaction networks were visualized using the “Search Tool for Recurring Instances of Neighboring Genes” (STRING 12.0 release 26 July 2023, European Molecular Biology Laboratory, Hinxton, England), setting the minimum required interaction score at 0.4 and hiding disconnected nodes [48].

## 3. Results

### 3.1. Productive Performance of Sows and Litter Growth

The productive performance of sows registered during the trial is reported in Table 2. From the entrance into the farrowing room until parturition, no differences were found among groups in terms of pre-farrowing period duration. On average, lactation lasted 28.50 ± 1.22 days, and no differences were found among the experimental groups. No differences were found among groups when considering the cumulative feed intake. Notably, farrowing time decreased (Table 2) in the ACTD1 and ACTD2 groups in comparison to CTR: ACTD1 sows registered a reduction of 25.36%, whereas ACTD2 sows evidenced a reduction of farrowing duration corresponding to 23.26% (*p* < 0.05).

Body weight variations throughout the trial were not statistically different among groups. Hence, body weight loss was only numerically higher for the ACTD1 and ACTD2 groups in comparison to the CTR group. Backfat thickness evaluation revealed no differences among groups at the entrance in the farrowing room or after parturition. However, both ACTD1 and ACTD2 evidenced a thinner backfat layer (Table 2) in comparison to the CTR group at the end of lactation, with reductions of 17.36% and 18.26% (*p* < 0.05).

Litter consistency and growth data are reported in Table 3. Analysis of litter consistency and viability parameters, including the total number of born, mummified, stillborn, and alive piglets, revealed no significant differences among the experimental groups. In turn, ACTD1 and ACTD2 litter weights at farrowing (Table 3) were higher and evidenced increases of 23.87% and 25.65% in comparison to the CTR group (*p* < 0.05). Moreover, the average body weight (Table 3) of ACTD1 and ACTD2 piglets improved to extents corresponding to 12.59% and 14.17% when compared to CTR piglets (*p* < 0.01). Following cross-fostering, the ACTD2 group displayed a similar trend to the average litter, and piglet weights were higher in comparison to those of CTR (*p* < 0.05). Between cross-fostering and the end of lactation, piglet mortality was significantly lower in ACTD1 litters compared to CTR (*p* < 0.05), whereas ACTD2 litters showed only a numerical difference when compared to CTR. However, the difference in terms of total weaned piglets among groups tended to be significant (*p* = 0.07), with ACTD1 and ACTD2 having 1.29 (+13.75%) and 1.17 (+12.47%) more piglets on average when compared to CTR. Moreover, both average litter and piglet weights at weaning were improved in ACTD1 and ACTD2 in comparison to CTR, with single animals registering increases of 9.89% and 12.46% on average (*p* < 0.01).

### 3.2. Plasma Pro-Inflammatory Cytokine Evaluation

**Figure 2:**
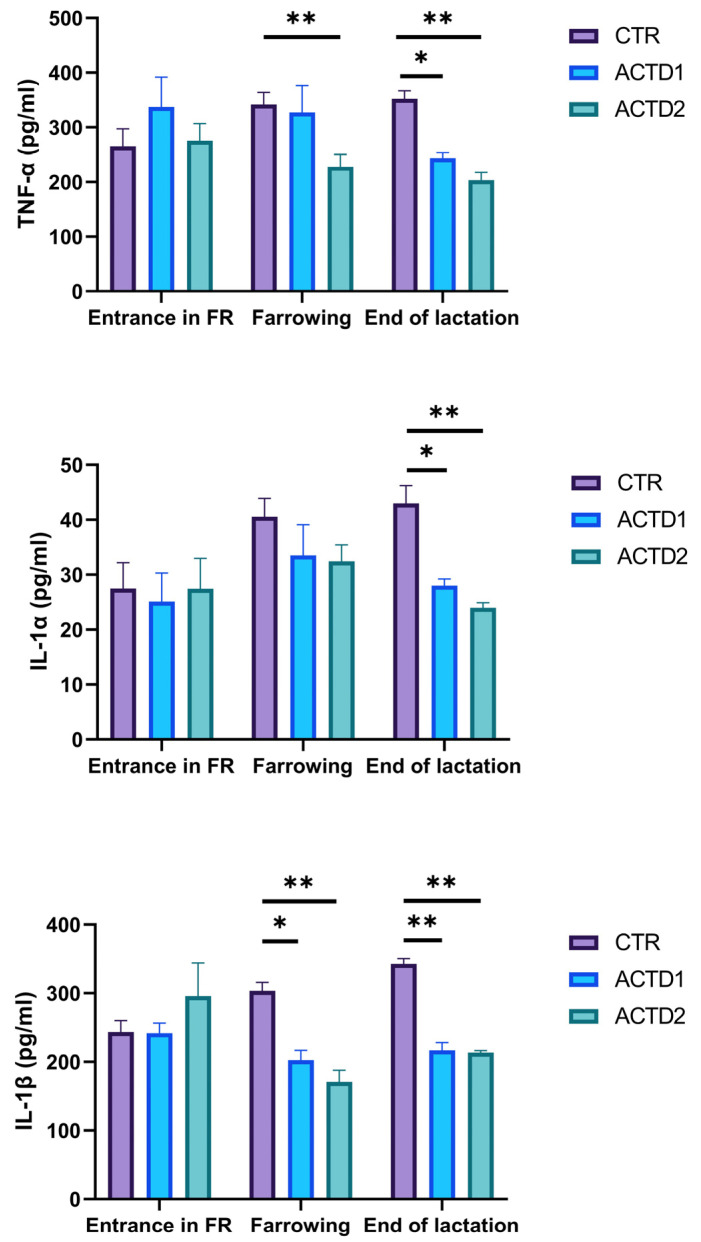
Tumor necrosis factor alpha (TNF-α), interleukin one alpha (IL-1α), and one beta (IL-1β) plasma levels quantified upon entrance into the farrowing room (FR), after farrowing and at the end of lactation. All the results are reported as mean ± SEM. Statistically significant differences are reported as follows: * = *p* < 0.05; ** = *p* < 0.01. Control (CTR): basal diet; treatment 1 (ACTD1): basal diet + 400 mg/kg of the premix providing 0.64 µg/kg of glycosylated calcitriol and 140 µg/kg of a combination of UA + OA triterpenes; treatment 2 (ACTD2): basal diet + 600 mg/kg of the premix providing 0.96 µg/kg of glycosylated calcitriol and 210 µg/kg of UA + OA triterpenes.

Pro-inflammatory cytokine results are shown in Figure 2. TNF-α was not influenced by the dietary treatments upon entrance into the farrowing room. After farrowing, ACTD2 sows evidenced a reduction in TNF-α when compared to CTR sows (Figure 2, *p* < 0.01).

In addition, TNF-α highlighted a reduction at the end of lactation in ACTD1 and ACTD2 sows in comparison to CTR ones (Figure 2, *p* < 0.05). On the other hand, it can be seen that IL-1α was not conditioned during the first two time points but was positively influenced by the administration of glycosylated calcitriol combined with natural triterpenes at the end of lactation, regardless of the dosage. Indeed, both ACTD1 and ACTD2 sows evidenced lower IL-1α levels in comparison to CTR (Figure 2, *p* < 0.05). On the other hand, IL-1β did not differ among groups upon entrance into the farrowing room. Nonetheless, ACTD1 and ACTD2 dietary treatments modulated IL-1β levels, favoring a reduction in the circulating pro-inflammatory biomarker after farrowing (Figure 2, *p* < 0.05). Notably, this positive trend was maintained at the end of lactation, as both the ACTD1 and ACTD2 groups evidenced reduced plasma concentrations of IL-1β in comparison to those of CTR (Figure 2, *p* < 0.01).

### 3.3. Plasma Parathormone and Calcitriol Evaluation

**Figure 3:**
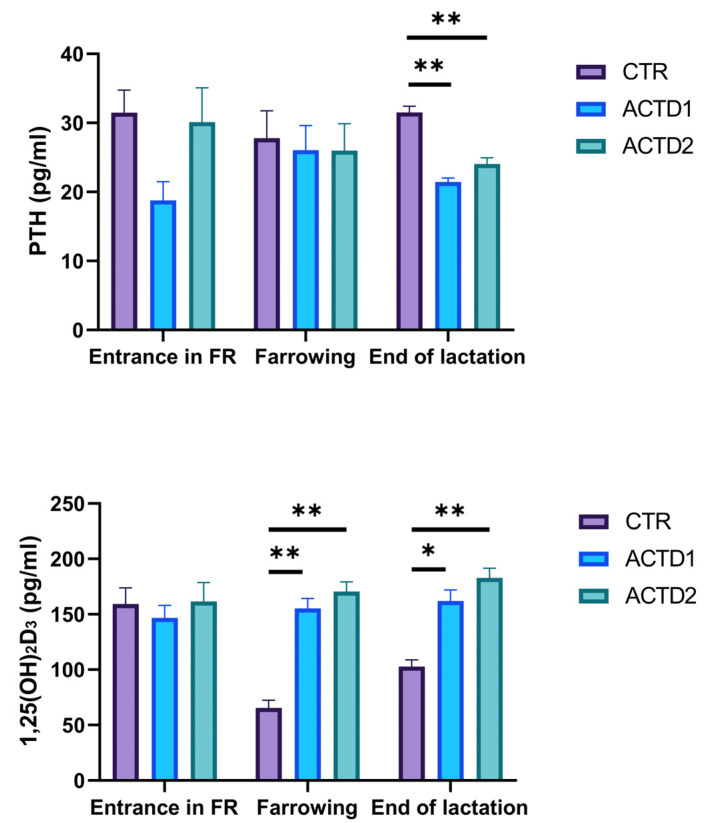
Parathormone (PTH) and calcitriol (1,25(OH)_2_D_3_) plasma levels quantified upon entrance into the farrowing room (FR), after farrowing, and at the end of lactation. All the results are reported as mean ± SEM. Statistically significant differences are reported as follows: * = *p* < 0.05; ** = *p* < 0.01. Control (CTR): basal diet; treatment 1 (ACTD1): basal diet + 400 mg/kg of the premix providing 0.64 µg/kg of glycosylated calcitriol and 140 µg/kg of a combination of UA + OA triterpenes; treatment 2 (ACTD2): basal diet + 600 mg/kg of the premix providing 0.96 µg/kg of glycosylated calcitriol and 210 µg/kg of UA + OA triterpenes.

As depicted by Figure 3, plasma PTH levels were not different among groups upon entrance into the farrowing room and after farrowing. However, lower PTH levels were detected in plasma samples of ACTD1 and ACTD2 sows at the end of lactation (Figure 3, *p* < 0.01). 1,25(OH)_2_D_3_ did not differ among experimental groups at trial start. Nonetheless, ACTD1 and ACTD2 sows evidenced an improved 1,25(OH)_2_D_3_ status both after farrowing (Figure 3, *p* < 0.01) and at the end of lactation (Figure 3, *p* < 0.05) in comparison to CTR, highlighting an opposite trend in relation to PTH levels.

### 3.4. Plasma Calcium and Phosphate Levels

**Figure 4:**
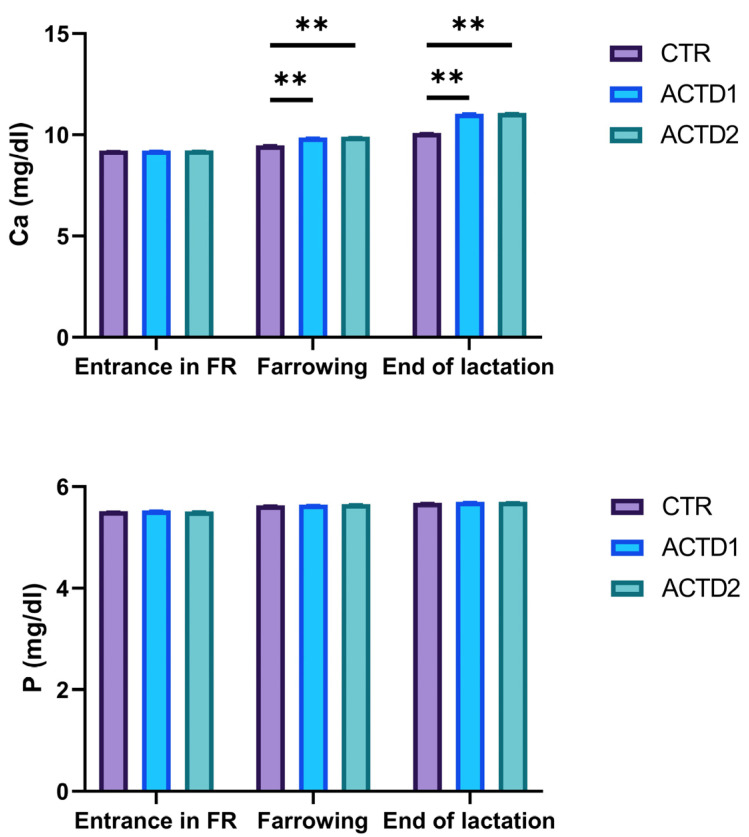
Calcium (Ca) and phosphate (P) plasma levels quantified upon entrance into the farrowing room (FR), after farrowing, and at the end of lactation. All the results are reported as mean ± SEM. Statistically significant differences are reported as follows: ** = *p* < 0.01. Control (CTR): basal diet; treatment 1 (ACTD1): basal diet + 400 mg/kg of the premix providing 0.64 µg/kg of glycosylated calcitriol and 140 µg/kg of a combination of UA + OA triterpenes; treatment 2 (ACTD2): basal diet + 600 mg/kg of the premix providing 0.96 µg/kg of glycosylated calcitriol and 210 µg/kg of UA + OA triterpenes.

As highlighted in Figure 4, plasma calcium levels were characterized by a lack of differences among groups upon entrance into the farrowing room. However, both dosages of glycosylated calcitriol combined with triterpenes positively influenced calcium levels in ACTD1 and ACTD2 in comparison to CTR, both after farrowing and at the end of lactation (Figure 4, *p* < 0.01). Conversely, no differences were found among groups in terms of phosphate plasma concentration upon entrance into the farrowing room, after farrowing, and at the end of lactation.

### 3.5. Serum Proteomic Profile

To explore the molecular effects of the dietary inclusion of glycosylated calcitriol combined with the triterpenes ursolic acid and oleanolic acid during the peripartum period, a label-free shotgun proteomic approach was applied to serum samples collected from sows at three physiological stages: upon entry into the farrowing room (A), after farrowing (B), and at the end of lactation (C).

**Figure 5:**
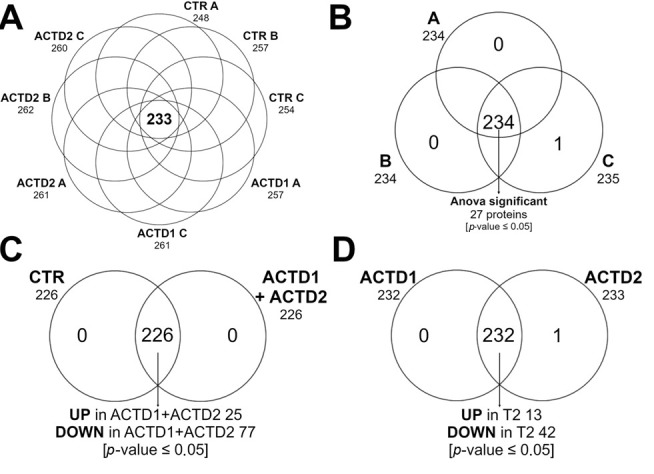
Venn diagrams of the proteins identified in the eight data sets (**A**) and in the comparisons: (**B**) A vs. B vs. C, (**C**) ACTD1+ ACTD2 vs. CTR and (**D**) ACDT2 vs. ACDT1. The proteomic analysis identified proteins common to all samples, as well as proteins exclusively expressed in each data set. PrG = protein group. Control (CTR): basal diet; treatment 1 (ACTD1): basal diet + 400 mg/kg of the premix providing 0.64 µg/kg of glycosylated calcitriol and 140 µg/kg of a combination of UA + OA triterpenes; treatment 2 (ACTD2): basal diet + 600 mg/kg of the premix providing 0.96 µg/kg of glycosylated calcitriol and 210 µg/kg of UA + OA triterpenes.

The total number of proteins identified and quantified within each group at each time point is shown in the Venn diagrams reported in Figure 5, A. As shown in the figure, the eight proteome conditions resulted in no proteins expressed exclusively and a total of 233 proteins common to all data sets, among which 100 were ANOVA-significant (*p* < 0.05).

To characterize proteomic differences induced by farrowing and the treatment in more detail, comparisons were performed between A vs. B vs. C, ACTD1+ ACTD2 vs. CTR, and ACDT2 vs. ACDT1. Each comparison showed proteins exclusively expressed, proteins statistically different, and proteins whose expression levels remained unchanged. The corresponding Venn diagrams are shown in Figure 5B, Figure 5C and Figure 5D respectively.

To investigate how the physiological stage affects the sow serum proteome independently of dietary treatment, a temporal comparison between the three time points—entry into the farrowing room (A), post-farrowing (B), and end of lactation (C)—was performed. This analysis aimed to characterize dynamic changes in protein abundance associated with the transition from late gestation to the end of lactation, a period characterized by intense metabolic, inflammatory, and endocrine adaptations.

The temporal comparison (A vs. B vs. C) identified a total of 234 proteins common to all data sets, among which 27 were differentially expressed across the three physiological stages (Figure 5B, *p* < 0.05). Functional enrichment analysis of these proteins revealed a significant overrepresentation of processes related to retinoid metabolism and transport, metabolism of fat-soluble vitamins and blood coagulation. In particular, changes in proteins associated with cholesterol transport and the lipoprotein metabolism network (APOA1, APOA4) were observed. Although apolipoprotein B (ApoB) is more heavily involved in the primary transport of dietary fats and vitamins (A, D, E, K), HDL particles and ApoA-I also play a role in transporting lipophilic substances, together with ApoA-4. These apolipoproteins act as carriers, facilitating the delivery of dietary, lipophilic compounds (such as fat-soluble vitamins) throughout the body [49]. Other pathways clearly enriched are acute-phase and coagulation response and immune regulation (APOA1, KNG1, FGB, ITIH4) (Supplementary Materials, Table S1), suggesting that the serum proteome without the inclusion of glycosylated 1,25(OH)_2_D_3_ and triterpenes undergoes a time-dependent modulation, characterized by activation of coagulation-related proteins after farrowing, followed by enhanced lipid transport and metabolic activity to support calcium homeostasis and lactation.

To assess the overall effect of the applied nutritional strategy, the combined groups receiving glycosylated calcitriol with triterpenes (ACDT1 and ACDT2) were pooled and compared to the control group (CTR). The comparison showed that the dietary inclusion of glycosylated 1,25(OH)_2_D_3_ with triterpenes markedly influenced the serum proteome of sows. A total of 102 proteins were differentially expressed, including 25 increased and 77 decreased in the ACDT1 + ACDT2 vs. CTR comparison (Figure 5C). Functional enrichment analysis using PANTHER on the differentially expressed proteins revealed significant enrichment of pathways related to coagulation cascades, platelet activation and hemostasis (Supplementary Materials, Table S1), which were more expressed in CTR in comparison to ACTD1 + ACTD2. Although there is no direct evidence regarding the combination of calcitriol and triterpenes, data from the literature report that both compounds independently modulate coagulation pathways, suggesting a potential synergistic anticoagulant and antiplatelet effect when combined.

Furthermore, to evaluate the existence of a dose-dependent response to the inclusion of glycosylated calcitriol combined with natural triterpenes, a direct comparison between the two supplemented groups (ACTD2 vs. ACTD1) was performed, aiming to identify proteins whose abundance varied proportionally with the increased dietary inclusion level of the tested compound (from 400 to 600 mg/kg). The comparison identified a total of 232 proteins common to all data sets, of which 58 were differentially expressed—45 increased and 13 decreased—as shown in Figure 5D. Functional enrichment analysis highlighted the downregulation of several proteins associated with cholesterol transport and lipoprotein remodeling, including apolipoproteins (APOA4; APOC3), as well as proteins associated with the complement and coagulation cascades in ACTD2 (Supplementary Materials, Table S1).

Given that lactating sows are particularly prone to calcium and vitamin D deficiencies, specific attention was given to proteins involved in calcium metabolism, vitamin D transport, and their known molecular interactors within the serum proteome across all experimental comparisons. To ensure that low-abundance but potentially relevant proteins were not excluded, this search was conducted without applying the 70% threshold previously used in the proteomic analyses, manually comparing the data sets with the proteins known to be involved in calcium signaling and the vitamin D metabolic network according to the KEGG PATHWAY Database.

The analysis identified only one protein directly associated with calcium signaling: ryanodine receptor 1 (RyR1). RyR1 is a calcium-release channel located on the sarcoplasmic reticulum membrane that mediates Ca^2+^ efflux into the cytosol during muscle contraction [50]. This protein was detected across all experimental groups and time points but did not show any quantitative variation. When focusing on the vitamin D metabolic network, several proteins known to participate in or interact with vitamin D-dependent processes were identified, showing modulation independent of both dosages and sampling time. The same behavior was observed for transport and binding proteins potentially involved in the delivery and activation of vitamin D metabolites (APOA1, APOA4 and FGB), suggesting that the administered compound effectively reached systemic circulation and may have engaged molecular targets downstream of vitamin D receptor activation.

**Figure 6:**
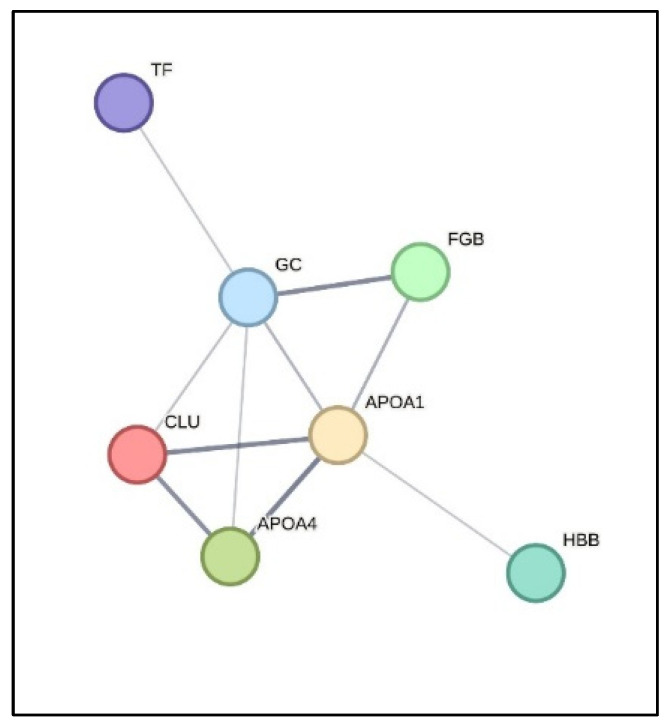
Network analysis of the proteins involved in vitamin D transport. Network analysis by String 12.0 (release 26 July 2023) of the proteins involved in vitamin D transport. Nodes represent identified proteins, and edges indicate predicted or known functional associations. Line thickness indicates the strength of evidence supporting each interaction (confidence interaction score 0.4). The network highlights functional links between vitamin D transport-related proteins and coagulation-associated proteins, including fibrinogen beta chain (FGB), serotransferrin (TF), and clusterin (CLU).

STRING network analysis (Figure 6) clearly showed that several proteins identified within the vitamin D transport network were functionally linked to coagulation-related proteins, including the fibrinogen subunit (FGB), serotransferrin (TF) and clusterin (CLU). These proteins were differentially expressed and mainly downregulated in the ACTD2 vs. ACTD1 and ACTD1 + ACTD2 vs. CTR comparisons (Supplementary Materials, Table S1), suggesting that glycosylated 1,25(OH)_2_D_3_ combined with natural triterpenes may attenuate coagulation cascade activity, most likely thanks to the enhanced bioavailability of active vitamin D. Indeed, this finding is consistent with evidence indicating that high-dose vitamin D dietary administration can exert anticoagulant effects by inhibiting blood clot formation, while vitamin D deficiency has been associated with an increased risk of thrombosis [51]. Such effects are believed to arise from vitamin D’s ability to regulate key coagulation proteins and to reduce systemic inflammation, thereby promoting vascular and hemostatic balance.

Regarding the dose–response effects, the higher dosage (ACTD2) appeared to reduce the expression of CLU, GC and TF, which may reflect a possible feedback regulatory mechanism in response to increased systemic availability of 1,25(OH)_2_D_3_. Vitamin D-binding protein (GC) is the primary carrier of circulating vitamin D metabolites and plays a central role in regulating their bioavailability and half-life in plasma. Previous studies have shown that variations in vitamin D status are associated with changes in GC levels, suggesting adaptive regulation of transport capacity [52]. Similarly, clusterin (CLU) functions as an extracellular chaperone involved in the transport and stabilization of lipophilic molecules and hormone–protein complexes, with expression responsive to systemic metabolic and hormonal changes [53]. Transferrin (TF), although primarily responsible for iron transport, is also recognized as a multifunctional protein participating in metabolic and inflammatory networks, with evidence of functional interplay between vitamin D status and iron metabolism [54,55].

Therefore, the coordinated downregulation of these transport-related proteins at a higher dosage may indicate a reduced requirement for carrier-mediated transport once adequate circulating levels of active vitamin D are achieved.

## 4. Discussion

Testing blends of natural active compounds under commercial farm conditions may represent an effective strategy for sustaining pig productivity and health during critical phases of the production cycle [56]. As reported above, the combination of glycosylated calcitriol with natural triterpenes has the potential to optimize 1,25(OH)_2_D_3_ uptake and bioavailability, affecting Ca absorption and sustaining the immune status of sows throughout late gestation and lactation. Therefore, the objective of the present study was to evaluate the effects of this combination and to explore the underlying mechanisms.

In the present study, no differences were detected among experimental groups when considering body weight variations and feed intake of sows. Previous evaluations evidenced a lack of effect on body weight variations in multiparous sows when supplementing alternative forms of vitamin D. In particular, Lauridsen et al. [18] reported similar BW changes between sows fed different forms of vitamin D (calcidiol and cholecalciferol) supplemented at different dosages. Likewise, Flohr et al. [57] tested increasing dietary vitamin D_3_ levels (1500, 3000, or 6000 IU/kg) in productive sows and observed no differences in body weight change or average daily feed intake throughout the trial. Therefore, it seems that regardless of the form of administration or the dosage, vitamin D is unable to modulate sow BW during late gestation and lactation. As per the triterpene component, natural extracts blend previously displayed limited effects in conditioning the BW development of multiparous sows. Specifically, Herve et al. [58] reported that a top-dressed blend of natural extracts supplemented from day 106 of gestation to the end of lactation did not affect the BW changes of multiparous sows. Consistent with these findings, our results indicate that dietary administration of glycosylated calcitriol combined with UA and OA triterpenes did not affect sow body weight modulation or feed intake from farrowing to the end of the lactation period, most likely because the feed was not offered ad libitum.

Flohr et al. [59] performed a dose–response study focused on the dietary administration of cholecalciferol. The authors reported that increasing cholecalciferol dosage had no effect on lactation backfat loss. In contrast, Lütke-Dörhoff et al. [60] reported that the dietary administration of 50 µg/kg of calcidiol can limit backfat loss during lactation. In the latter case, the authors discussed how these changes could be addressed by the possible effects of calcidiol on body composition. In addition, the previously mentioned study by Herve et al. [58] showed that blended natural extracts are unable to modulate backfat loss during lactation. Nonetheless, in the present study, ACTD1 and ACTD2 sows were characterized by a lower backfat thickness in comparison to CTR animals at the end of lactation. These conflicting results may be explained by the presence of litters that tended to be more consistent and heavier in the ACTD1 and ACTD2 farrowing crates. Indeed, it is noteworthy that sows characterized by heavier litters could be subjected to a higher mobilization of energy to accommodate milk demand [61].

An excessive farrowing duration may expose piglets to hypoxia, reducing litter livability and growth during lactation [62]. In the present study, ACTD1 and ACTD2 sows were characterized by a shorter farrowing time. In a previous study performed by Jahn et al. [63], the administration of calcitriol glycosides in gestating sows raised in a free-farrowing system reduced farrowing duration. Further studies should focus on vitamin D receptors expressed in reproductive organs to clarify the modes of action of calcitriol glycosides in reducing farrowing duration [64], also considering the possible effects of the triterpene component. Indeed, herbal extract blends have been reported to reduce farrowing duration [65]. This effect can be related both to the enhancement in hemoglobin concentration and energy metabolism [66,67] but deserves additional evaluations. In the same vein, proteomic analyses revealed modulation of coagulation cascades and hemostasis pathways in supplemented sows, suggesting variation in vascular homeostasis during parturition.

The administration of alternative forms of vitamin D has been shown to be effective in conditioning litter livability and growth. In particular, Upadhaya et al. [67] evidenced that the dietary administration of 50 µg/kg of calcidiol in a basal diet containing 1500 IU of vitamin D_3_ to sows positively influenced the average daily gain and weaning BW of piglets. Similarly, Zhang et al. [68] reported that the dietary administration of 50 µg/kg of calcidiol in multiparous sows enhanced litter growth during the first 21 days of lactation compared with a basal diet containing 2000 IU/kg of vitamin D_3_. Moreover, there is an increasing number of studies reporting that plant-derived compounds can improve litter growth performance when administered to dams. For instance, piglets born from sows fed with oregano essential oil, which is rich in carvacrol and thymol, evidenced improved growth performance during the first week of life [69]. In addition, Wang et al. [70] administered 1.0 g/kg of an herbal extract composed of 50% of *S. baicalensis* and 30% *L. japonica* extracts during late gestation and lactation. The authors reported higher birth weights and livability, which further resulted in improved weaning weights. In a study performed by Santos et al. [71], a blend of diverse essential oils, primarily composed of menthol (1.80 mg/kg), trans-anethole (0.76 mg/kg), and thymol (0.41 mg/kg), added to a lactation diet containing 0.03% calcidiol, positively influenced the average weight of piglets at weaning. These findings suggest that dietary administration of 1,25(OH)_2_D_3_ with natural triterpenes in sows during late gestation and lactation may indirectly enhance piglet growth by improving intrauterine development, colostrum, and milk supply, likely as a consequence of the improved metabolic and immune status of the sow. Indeed, the proteome profile also indicated modulation of lipid-transport and nutrient-carrier proteins, which may reflect enhanced nutrient mobilization from dam to litter, supporting improved piglet growth performance. Nonetheless, given the limited number of subjects involved in the study, the results related to productive performance and litter growth should be interpreted with caution and warrant further evaluation in larger cohorts.

Vitamin D is largely associated with immunomodulatory properties. As reported by Asgharpour et al. [72], various cellular components of the immune system, including CD4+ and CD8+ T cells, B cells, neutrophils, macrophages, and dendritic cells, are capable of expressing vitamin D receptors (VDRs). 1,25(OH)_2_D_3_ can interact with VDRs, and these mechanisms influence immunomodulatory pathways. In particular, Motamed et al. [73] highlighted that 1,25(OH)_2_D_3_ levels tend to increase in late pregnancy due to enhanced intestinal absorption. In addition, the elevated circulating levels of calcitriol could be associated with a downregulation of pro-inflammatory cytokines. As reported above, the triterpene components of the tested solution may also play a key role in immune modulation. The dietary administration of plant extracts can reduce circulating TNF-α and IL-1β in gestating and lactating sows [74]. On the other hand, studies specifically reporting IL-1α variations due to different vitamin D forms and/or triterpenes are less consistent in the available literature. An exception is represented by a previous study focused on suckling piglets performed by Becker et al. [75]. The authors tested the same solution evaluated in the present study and found a decrease in TNF-α, IL-1α and IL-1β that was driven by time factors. Specifically, the latter case was related to the authors increasing the feed intake; they also discussed the possible role of the environment in the development of the circulating cytokine profile of suckling piglets. Based on these findings, it is reasonable to hypothesize that 1,25(OH)_2_D_3_ glycosides combined with triterpenes could bring notable immunomodulatory properties, especially in relation to circulating pro-inflammatory biomarkers. Proteomic data further support this hypothesis, showing differential expressions of inflammation-related proteins such as SERPINAC1 and complement/hemostatic factors (see Supplementary Materials, Table S1). These pathways, together with reduced circulating pro-inflammatory cytokines, suggest that the dietary inclusion of glycosylated 1,25(OH)_2_D_3_ with natural triterpenes may attenuate systemic inflammatory tone and modulate innate immune pathways during lactation. However, further research is needed to strengthen the presented hypothesis.

A decline in blood calcium concentration stimulates the parathyroid glands to secrete parathyroid hormone (PTH). Hence, PTH acts to restore calcium homeostasis by reducing renal calcium excretion, enhancing the renal synthesis of 1,25(OH)_2_D_3_, which in turn increases intestinal Ca absorption [76]. Once Ca reaches prominent levels, PTH secretion falls following negative feedback regulated by calcium-sensing receptors (CaSRs), which brings limitations towards 1,25(OH)_2_D_3_ renal synthesis [77]. In pigs, the dietary administration of 1,25(OH)_2_D_3_ glycosides stimulates Ca and P intestinal absorption [78,79]. In the present study, ACTD1 and ACTD2 sows displayed higher plasma concentrations of Ca after farrowing and at the end of lactation. Concerning the latter time point, ACTD1 and ACTD2 sows were also characterized by lower PTH levels. In the present study, no variations in terms of P levels were detected among groups. Therefore, the reduction in PTH likely reflects a fine endocrine adjustment secondary to slightly increased calcemia. Indeed, the absence of variation in plasma phosphates suggests that in this study, the primary mechanism of action was not marked stimulation of intestinal mineral absorption but rather a mild endocrine adjustment and systemic modulation.

In more detail, the administration of glycosylated 1,25(OH)_2_D_3_ resulted in lower circulating PTH concentrations, accompanied by increased plasma calcium and calcitriol levels, while plasma phosphate remained unchanged. This pattern supports the tight regulation of phosphate homeostasis through the vitamin D–PTH axis. Although calcitriol enhances intestinal absorption of both calcium and phosphate, the improved calcium status likely contributed to PTH suppression, thereby limiting renal phosphate retention. Consequently, compensatory hormonal and renal mechanisms maintained stable plasma phosphate concentrations despite active vitamin D administration [21,79,80].

Overall, the supplement promoted a significant systemic anti-inflammatory modulation accompanied by a mild endocrine adjustment of the Ca–PTH–vitamin D axis, without direct evidence of a relevant change in intestinal mineral absorption. Therefore, the observed productive and reproductive improvements appear to be more associated with immune and vascular modulation than with a primary effect on mineral homeostasis.

In the same vein, proteomics did not reveal modulation of the main calcium-regulatory proteins in plasma. However, the identification of vitamin D-interacting proteins across treatments indicates that the supplemented compound was effectively absorbed and reached systemic circulation. The downregulation of CLU, GC and TF in ACTD2 compared with ACTD1 suggests a dose-dependent regulation of vitamin D transport and binding processes, which could be associated with the higher bioavailability of the active metabolite. Furthermore, STRING-based network analysis (see Figure 6) indicated that several vitamin D-interacting proteins, such as clusterin, serotransferrin, and the fibrinogen subunit, were functionally connected to coagulation pathways. Their downregulation in groups receiving the higher dosage of glycosylated 1,25(OH)_2_D_3_ suggests that improved vitamin D availability may contribute to a moderated coagulative response, consistent with reported anticoagulant and anti-inflammatory actions of vitamin D.

## 5. Conclusions

In conclusion, dietary supplementation with glycosylated 1,25(OH)_2_D_3_ combined with natural triterpenes during late gestation and lactation did not influence sow body weight or feed intake, in line with previous evidence on alternative vitamin D forms under controlled feeding conditions. However, the treatment was associated with reduced farrowing duration and improved litter performance.

The increase in circulating calcium, accompanied by lower parathormone concentrations, without changes in plasma phosphate, indicates an endocrine modulation of mineral homeostasis rather than a marked stimulation of mineral absorption. The latter effects may be particularly relevant in first- and second-parity sows, which experience greater calcium demands due to ongoing skeletal development and, therefore, deserve targeted investigation.

The observed reduction in pro-inflammatory cytokines, supported by proteomic evidence of modulation of inflammation- and hemostasis-related pathways, suggests the effective bioavailability of the supplemented compounds and systemic immunomodulatory action. Overall, improved endocrine balance and reduced inflammatory tone may have contributed to supporting sow resilience during critical productive phases. Despite these promising results, the limited availability of comparable studies warrants caution. Further large-scale trials under commercial conditions are needed to confirm these findings and assess their practical relevance in contemporary pig production systems.

## Figures and Tables

**Table 1:** Ingredients and nutrients analyzed in composition of the basal diet (as-fed basis, %).

Ingredients, % as Fed	Gestation	Lactation
Corn	17.46	23.85
Barley	20.75	19.55
Wheat bran	22.80	13.00
Bakery by-products	9.20	10.80
Distillers	12.50	8.00
Sugar beet pulp	4.60	6.925
Soybean oil	1.30	1.30
Fish meal	-	1.625
Grounded linseed	-	1.625
Yogurt	-	1.625
Dehulled soybean meal	5.00	5.00
Extruded soybean meal	3.00	3.00
Limestone	1.60	1.05
Dicalcium phosphate	0.20	0.90
Salt	0.40	0.40
L-lysine, 70%	0.15	0.11
Threonine	0.04	0.02
Mineral and vitamin premix ^1^	1.00	1.00
**Analyzed nutrient content, % as fed**		
Crude protein	14.85	18.21
Ether extract	4.22	6.18
Crude fiber	5.37	5.88
Ash	5.92	5.98
Calcium	0.80	1.20
Phosphorus	0.57	0.68
Lysine (calculated)	0.90	1.07
Methionine (calculated)	0.20	0.22

^1^ Providing (per kg of complete diet): vitamin A, 10,000 IU; vitamin D3, 1800 IU; vitamin E, 48 IU; vitamin K3, 1.5 mg; riboflavin, 6 mg; niacin, 40 mg; biotin, 0.2 mg; d-pantothenic, 17 mg; folic acid, 2 mg; choline, 166 mg; vitamin B6, 2 mg; and vitamin B12, 28 mg. Fe (as FeSO_4_), 90 mg; Cu (as CuSO_4_), 15 mg; Zn (as ZnSO4), 50 mg; Mn (as MnO_2_), 54 mg; I (as KI), 0.99 mg; Se (as Na2SeO3), 0.25 mg; phytase, 476 FTU/kg.

**Table 2:** Productive performance of sows registered from the entrance into the farrowing room until the end of lactation.

Parameters	Experimental Groups ^1^			Statistic	
**Sows**	**CTR**	**ACTD1**	**ACTD2**	**SEM**	***p*** **-Value**
Number of sows	8	8	8	-	-
**Average Duration, Days**					
Pre-farrowing period	4.13	4.88	4.53	0.61	0.48
Lactation	28.88	28.13	28.50	0.61	0.48
**Cumulative Feed Intake, kg** **^2^**					
Entrance into farrowing room–Parturition	11.55	13.65	12.60	1.72	0.49
Parturition–End of lactation	198.70	192.70	195.70	4.92	0.49
**Farrowing Time, minutes**	236.35 **^a^**	176.42 **^b^**	181.38 **^b^**	14.44	<0.05
**Sows Body Weight, kg**					
Entrance in farrowing room	318.10	322.06	320.10	13.69	0.95
End of lactation	271.31	259.44	258.25	15.49	0.66
Body weight loss	46.69	62.63	61.85	9.14	0.17
**Average Backfat Thickness, mm**					
Entrance into farrowing room	13.76	14.98	14.08	1.41	0.67
Farrowing	11.76	12.37	12.80	1.09	0.64
End of lactation	11.06 **^a^**	9.14 **^b^**	9.04 **^b^**	0.69	<0.05

^1^ Control (CTR): basal diet; treatment 1 (ACTD1): basal diet + 400 mg/kg of the premix providing 0.64 µg/kg of glycosylated calcitriol and 140 µg/kg of a combination of UA and OA triterpenes; treatment 2 (ACTD2): basal diet + 600 mg/kg of the premix providing 0.96 µg/kg of glycosylated calcitriol and 210 µg/kg of UA + OA triterpenes. All the results are reported as mean ± SEM. Different letters within the same row indicate statistically significant differences (a, b = *p* < 0.05). ^2^ Cumulative feed intake was based on dry feed.

**Table 3:** Litter consistency and growth performance throughout the trial.

Parameters	Experimental Groups ^1^			Statistics	
**Farrowing**	**CTR**	**ACTD1**	**ACTD2**	**SEM**	***p*** **-Value**
Total born, n	15.13	16.13	16.50	2.06	0.79
Mummified, n	0.50	0.25	0.75	0.44	0.54
Stillborn, n	1.75	1.25	1.00	0.79	0.64
Total alive, n	12.88	14.63	14.75	1.44	0.36
Average litter weight, kg	16.84 **^b^**	20.86 **^a^**	21.16 **^a^**	1.78	<0.05
Average piglet weight, kg	1.27 **^B^**	1.43 **^A^**	1.45 **^A^**	0.04	<0.01
**Cross-fostering** **^2^**					
Total piglets after cross-fostering, n	10.63	10.80	10.80	0.47	0.95
Average litter weight, kg	18.35 **^b^**	20.60 **^ab^**	21.53 **^a^**	1.09	<0.05
Average piglet weight, kg	1.73 **^b^**	1.92 **^ab^**	2.00 **^a^**	0.09	<0.05
**End of lactation**					
Piglet mortality, n	1.25 **^a^**	0.13 **^b^**	0.25 **^ab^**	0.41	<0.05
Total weaned piglets, n	9.38	10.67	10.55	0.57	0.07
Average litter weight, kg	61.69 **^B^**	77.08 **^A^**	78.11 **^A^**	4.22	<0.01
Average piglet weight, kg	6.58 **^B^**	7.23 **^A^**	7.40 **^A^**	0.23	<0.01

^1^ Control (CTR): basal diet; treatment 1 (ACTD1): basal diet + 400 mg/kg of the premix providing 0.64 µg/kg of glycosylated calcitriol and 140 µg/kg of a combination of UA + OA triterpenes; treatment 2 (ACTD2): basal diet + 600 mg/kg of the premix providing 0.96 µg/kg of glycosylated calcitriol and 210 µg/kg of UA + OA triterpenes. All the results are reported as mean ± SEM. Different letters within the same row indicate statistically significant differences (A, B = *p* < 0.01; a, b = *p* < 0.05). ^2^ Data refer to day 7 of life, representing the end of the cross-fostering period in compliance with the trial’s farm practices.

## Data Availability

The raw data supporting the conclusions of this article will be made available by the authors upon request.

## Supplementary Materials

The following supporting information can be downloaded at https://www.mdpi.com/article/10.3390/vetsci13030246/s1. Table S1: List of reactome pathways identified by Panther classification analysis and enriched in the proteins differentially expressed in the physiological stage comparisons (A vs. B vs. C), ACTD1+ ACTD2 vs. CTR and ACDT2 vs. ACDT1.
