# Supplementary material for: Effects of a Glycosylated Form of Active Vitamin D Combined with Natural Triterpenes on Sow Productive Performance, Mineral Homeostasis, Immune Biomarkers and Serum Proteome
Source: Vet Sci. 2026 Mar 5;13(3):246. doi: 10.3390/vetsci13030246 (PMC13030638; doi:10.3390/vetsci13030246)
Supplement: Supplementary file 1 [file vetsci-13-00246-s001.zip › vetsci-4117361-supplementary.pdf]

Table S1. List of Reactome pathways identified by Panther classification analysis and enriched in the proteins differentially expressed in the physiological stage comparisons (A vs. B vs. C), ACTD1+ ACTD2 vs. CTR and ACDT2 vs. ACDT1.

Reactome pathways	Counts	Fold Enrichment	Raw P-value	FDR	Gene
**A vs B vs C comparison**					
Retinoid metabolism and transport	3	> 100	6.48E-08	1.10E-04	APOA1, APOA4, TTR
Metabolism of fat-soluble vitamins	3	95.9	8.11E-08	6.90E-05	APOA1, APOA4, TTR
Visual phototransduction	3	54.4	8.24E-07	4.68E-04	APOA1, APOA4, TTR
Platelet degranulation	4	34.7	5.00E-06	2.13E-03	APOA1, KNG1, FGB, ITIH4
Response to elevated platelet cytosolic Ca2+	4	33.5	5.80E-06	1.97E-03	APOA1, KNG1, FGB, ITIH4
Platelet activation, signaling and aggregation	4	17.9	6.81E-05	1.16E-02	APOA1, KNG1, FGB, ITIH4
**ACTD1+ACTRD2 vs CTR comparison**					
Common Pathway of Fibrin Clot Formation	3	> 100	9.27E-07	3.94E-04	FGB, SERPINC1, PROC
Intrinsic Pathway of Fibrin Clot Formation	3	> 100	1.14E-06	3.88E-04	FGB, SERPINC1, PROC
Formation of Fibrin Clot (Clotting Cascade)	4	> 100	5.67E-08	9.64E-05	A2M, FGB, SERPINC1, PROC
Platelet degranulation	4	36.2	2.45E-07	2.09E-04	A2M, FGB, CLU, TF
Response to elevated platelet cytosolic Ca2+	4	34.8	2.96E-07	1.68E-04	A2M, FGB, CLU, TF
Platelet activation, signaling and aggregation	4	18.6	6.50E-06	1.38E-03	A2M, FGB, CLU, TF
Hemostasis	6	10.7	2.97E-06	8.42E-04	A2M, FGB, SERPINC1, PROC, CLU, TF
Regulation of Insulin-like Growth Factor (IGF) transport and uptake by Insulin-like Growth Factor Binding Proteins (IGFBPs)	4	31.0	8.09E-06	1.53E-03	SERPINC1, C3, PROC, TF
**ACTD2 vs ACTD1**					
Transport of gamma-carboxylated protein precursors from the endoplasmic reticulum to the Golgi apparatus	4	> 100	3.49E-05	4.24E-03	PROS1, PROC
Formation of Fibrin Clot (Clotting Cascade)	8	60.44	4.99E-04	3.03E-02	PROS1, PROT
Chylomicron remodeling	2	> 100	4.49E-05	5.09E-03	APOC3, APOA4
Chylomicron assembly	2	> 100	4.49E-05	4.49E-03	APOC3, APOA4
Terminal pathway of complement	2	53.9	5.95E-04	2.60E-02	CLU, C3
Platelet degranulation	4	39.3	7.74E-23	1.32E-19	PROS1, CLU, AHSH, TF
Retinoid metabolism and transport	5	97.38	7.65E-08	1.30E-04	APOC3, TTR, APOA4
Regulation of Insulin-like Growth Factor (IGF) transport and uptake by Insulin-like Growth Factor Binding Proteins (IGFBPs)	4	35.77	4.47E-06	9.51E-04	C3, AHSG, PROC, TF
Neutrophil degranulation	4	10.71	8.76E-05	7.46E-03	TTR, C3, AHSG, B2M
